# Workplace Environment and Lifestyle Factors Associated With Hyperuricemia Risk Among Male Manufacturing Workers: A Cross-Sectional Study at a Single Company in Japan

**DOI:** 10.7759/cureus.111627

**Published:** 2026-06-27

**Authors:** Yutaro Takahashi, Rara Tanojiri, Ryota Kumakura, Rie Okamoto, Shihua Yu, Shizuko Omote

**Affiliations:** 1 Health Sciences, Institute of Medical, Pharmaceutical and Health Sciences, Kanazawa University, Kanazawa, JPN

**Keywords:** breakfast skipping, company cafeteria use, exercise, hyperuricemia, lifestyle factors, male workers, manufacturing sector, occupational health, smoking, workplace factors

## Abstract

Background

Hyperuricemia affects many men in Japan, with the highest prevalence noted among working-age men. Elevated serum uric acid is independently associated with hypertension, metabolic syndrome, cardiovascular disease, and impaired renal function. However, uric acid measurement is not mandated in routine occupational health checkups, making early hyperuricemia detection and intervention inadequate in workplace settings. Moreover, a limited number of studies have comprehensively examined both workplace environment and lifestyle factors associated with serum uric acid levels in a single company.

Methods

This cross-sectional study enrolled male workers at a single manufacturing company in Japan (≥1,000 employees, no night shifts). Of the 872 workers who underwent periodic health checkups in June 2025, 613 were included after excluding women, technical intern trainees, those receiving hyperuricemia treatment, and those whose questionnaire data were incomplete (effective response rate: 70.3%). Participants were classified into normal and high uric acid groups (serum uric acid levels <7.0 and ≥7.0 mg/dL, respectively). Workplace environment factors (job type, lunch type, and fluid intake) and lifestyle factors (breakfast skipping, exercise habits, smoking, alcohol consumption, and sleep) assessed using a questionnaire, along with clinical data from health checkups, were compared between the groups. Multivariable logistic regression analysis with stepwise variable elimination was used to identify factors independently associated with elevated serum uric acid.

Results

The mean participant age was 39.9 ± 12.6 years, and 126 (20.6%) were classified into the high uric acid group. Compared with the normal group, the high uric acid group showed significantly higher body weight, body mass index, waist circumference, blood pressure, total cholesterol, low-density lipoprotein cholesterol, liver function markers (aspartate aminotransferase, alanine aminotransferase (ALT), and gamma-glutamyl transferase), and creatinine, as well as a significantly higher metabolic syndrome prevalence. Multivariable logistic regression identified smoking (odds ratio or OR: 1.861, 95% confidence interval or CI: 1.124-3.080), skipping breakfast three or more times weekly (OR: 1.684, 95% CI: 1.036-2.737), and ALT (OR: 1.035, 95% CI: 1.022-1.048) as significant risk-increasing factors, and company cafeteria use (OR: 0.528, 95% CI: 0.304-0.919), regular exercise (OR: 0.501, 95% CI: 0.288-0.871), age (OR: 0.952, 95% CI: 0.929-0.976), and estimated glomerular filtration rate (OR: 0.969, 95% CI: 0.950-0.988) as significant risk-reducing factors.

Conclusion

Company cafeteria use as a workplace environment factor, and exercise habits, breakfast skipping, and smoking as lifestyle factors, were independently associated with hyperuricemia risk among male workers. These findings suggest that promoting company cafeteria use, supporting regular exercise habits, encouraging breakfast consumption, and providing smoking cessation support are specific priorities for reducing hyperuricemia risk among male manufacturing workers. Given that the high uric acid group was relatively young, proactive uric acid screening in periodic health checkups and early interventions targeting younger workers are important priorities for workplace health promotion.

## Introduction

Hyperuricemia, defined as a serum uric acid level exceeding 7.0 mg/dL regardless of sex or age, affects approximately 20-25% of men in Japan [1]. In 2022, approximately 1.23 million of the 1.31 million outpatients receiving gout treatment in Japan were male [2], underscoring the substantial disease burden of hyperuricemia and gout among working-age men. Despite this burden, uric acid measurement is not mandated in the occupational health checkup required under Japan's Industrial Safety and Health Act, nor is the specific health checkup for metabolic syndrome mandated under the Act on Assurance of Medical Care for Elderly People [3], making systematic early detection and intervention in workplace settings inadequate.

Beyond gout, hyperuricemia is independently associated with a broad spectrum of cardiometabolic conditions. Elevated serum uric acid is an independent predictor of metabolic syndrome [4], doubling the risk of its development [5]. Each 1 mg/dL increase in the uric acid level increases hypertension prevalence by approximately 20% [6], and asymptomatic hyperuricemia is a predictor of incident hypertension and chronic kidney disease [7]. Elevated uric acid is also associated with increased cardiovascular and mortality risks [8], hepatic steatosis and liver dysfunction [9], and impaired renal function, serving as an independent risk factor for chronic kidney disease progression [10]. These findings position uric acid as a key biomarker for the comprehensive management of lifestyle-related diseases.

Among the factors associated with hyperuricemia, both workplace environmental and lifestyle factors have been reported to play a role. Shift and night work disrupt circadian rhythms and increase the risk of hyperuricemia [11,12]. High dietary fat intake in workplace meals [13] and consumption of sugar-sweetened beverages at work [14] may also increase uric acid levels through obesity-related mechanisms, while prolonged sedentary behavior independently increases hyperuricemia risk [15]. High-intensity physical labor may increase uric acid levels through accelerated ATP catabolism and dehydration [16]. Gout resulting from hyperuricemia is also associated with increased worker absenteeism and reduced work productivity [17]. Excessive intake of purine-rich foods and fructose-containing beverages promotes uric acid synthesis [14,18], and alcohol, particularly beer, is strongly associated with elevated uric acid and increased gout risk [19]. Regular moderate aerobic exercise lowers uric acid through improvements in body weight and insulin sensitivity, whereas high-intensity short-duration exercise has the opposite effect [18]. Poor sleep quality and short sleep duration are also associated with elevated uric acid levels [20]. Although the association between smoking and uric acid has not been fully elucidated, data from recent large-scale epidemiological research suggest a positive association [21].

However, a limited number of studies have comprehensively examined both workplace environment and lifestyle factors in a single company. Multi-company studies risk confounding by organizational factors such as differing health management systems and workplace cultures. A single-company design minimizes such heterogeneity and enables more precise evaluation of the influence of each factor on uric acid levels. In Japan, many medium- and large-sized companies have an on-site company cafeteria as a standard employee welfare benefit, providing a workplace food environment that may systematically shape employees' dietary habits; however, this factor has not received sufficient research attention previously. Furthermore, existing studies on workers and uric acid have predominantly focused on specific occupational exposures such as shift work, and comprehensive within-company assessments of both workplace environment and lifestyle determinants remain scarce. This study aimed to address the following research question: Which workplace environmental and lifestyle factors are independently associated with hyperuricemia risk among male workers at a single manufacturing company in Japan? The findings are intended to inform the development of workplace health promotion strategies tailored to the occupational context.

## Materials and methods

Study design

This cross-sectional study was conducted in June 2025 among full-time male workers at a single manufacturing company (Company B) in Prefecture A, Japan, who had undergone their annual periodic health checkup.

Participants

Company B is a manufacturing company with 1,000 or more employees operating without night shifts. Of the 872 workers who underwent the health checkup, 815 provided written informed consent. The following individuals were excluded: women (because estrogen promotes renal uric acid excretion, making sex-combined analysis inappropriate), technical intern trainees (because their cultural backgrounds and lifestyle habits differ substantially from those of Japanese workers), individuals receiving pharmacological treatment for hyperuricemia (because medication may not reflect true uric acid levels), and those whose questionnaire data were incomplete. After applying these exclusion criteria, 613 participants were included in the final analysis (effective response rate: 70.3%).

Data collection

The questionnaire was developed based on factors previously reported to be associated with serum uric acid levels, including dietary habits, alcohol consumption, exercise, smoking, sleep, and workplace environment [11-16,18-21]. Questionnaire packets, including study information sheets and consent forms, were distributed alongside health checkup materials. Completed questionnaires and consent forms were collected by the researcher on the day of the health checkup and digitized at Company B. Company B then linked the digitized questionnaire data with health checkup results (clinical laboratory values and medical interview data) after removing personal identifiers and provided the de-identified dataset to the researcher. The periodic health checkup was conducted on-site at Company B and included a medical interview, anthropometric measurements, urinalysis, blood tests, physical examination, and electrocardiography.

Measures

Questionnaire Data

The questionnaire collected basic demographic information including sex and household composition ( nine categories). Workplace environmental variables included job type (white-collar: sales, clerical, and design; blue-collar: primarily factory work), years of service (years), and daily working hours (h). Workplace habits assessed included lunch type (seven categories: company cafeteria, supermarket, convenience store, restaurant, self-prepared bento, family-prepared bento, or skipping lunch), frequency of company cafeteria use among cafeteria users (times/week), and type of beverage consumed during work (five categories). Post-work habits assessed included frequency of alcohol consumption (three categories) and type of alcoholic beverage consumed (nine categories).

Periodic Health Checkup Data

Anthropometric measurements included height, body weight, body mass index (BMI; calculated as weight in kilograms divided by height in meters squared), waist circumference, systolic blood pressure, and diastolic blood pressure. Clinical laboratory values included liver function markers (aspartate aminotransferase (AST), alanine aminotransferase (ALT), and gamma-glutamyl transferase (γ-GTP)), serum lipids (total cholesterol, low-density lipoprotein (LDL) cholesterol, and high-density lipoprotein (HDL) cholesterol), serum uric acid, renal function markers (creatinine and estimated glomerular filtration rate (eGFR)), and glycated hemoglobin (HbA1c). Medical interview data included medical history, current medication use (antihypertensive, hypoglycemic, and lipid-lowering agents), weight gain of ≥10 kg since the age of 20 years, regular exercise habits (defined as engaging in exercise of at least moderate intensity for ≥30 min per session, ≥2 sessions per week, for ≥1 year), eating dinner within two hours of bedtime three or more times per week, snacking habits, skipping breakfast three or more times per week, sleep adequacy, and smoking status (current, former, or never).

Statistical analysis

Participants were classified into normal and high uric acid groups (serum uric acid level: <7.0 and ≥7.0 mg/dL, respectively). Although the Japan Society of Gout and Uric & Nucleic Acids [1] defines hyperuricemia as a serum uric acid level exceeding 7.0 mg/dL, the present study adopted 7.0 mg/dL as the cutoff value.

Descriptive statistics were first computed for all participants. Between-group comparisons were performed using Student's t-test and Pearson's chi-squared test for continuous and categorical variables, respectively. Variables with p<0.1 in the univariable analyses were entered as candidate variables for multivariable logistic regression analysis, as a liberal threshold was adopted to avoid premature exclusion of potential confounders. Although age did not meet the p<0.1 threshold in the univariable analysis, it was included as a candidate variable given its well-established role as a confounding factor for hyperuricemia.

Prior to the multivariable logistic regression analysis, variance inflation factors (VIF) were examined among candidate variables to assess multicollinearity. To minimize multicollinearity, BMI was selected over body weight (VIF=8.916) and waist circumference (VIF=10.848) as the measure of obesity, and eGFR was selected over creatinine (VIF=7.683) as the measure of renal function. Although the VIF values for systolic and diastolic blood pressure were within acceptable range, only systolic blood pressure was entered to avoid redundancy between conceptually related variables. All variables entered in the multivariable model had VIF values below five (maximum VIF=2.351), confirming acceptable levels of multicollinearity.

Multivariable logistic regression analysis with stepwise variable elimination was then conducted, with membership in the high uric acid group as the dependent variable. For the regression analysis, smoking status was recoded as one (current or former smoker) vs. zero (never smoker) to capture cumulative smoking exposure, and metabolic syndrome status was recoded as one (met criteria or borderline) vs. zero (did not meet criteria). All analyses were performed using IBM SPSS Statistics for Windows, Version 29 (Released 2022; IBM Corp., Armonk, New York, United States), with a two-sided significance level of 0.05.

Ethics

This study was approved by the Medical Ethics Review Committee of Kanazawa University (approval number: 111212). All participants provided written informed consent after receiving a written explanation of the study purpose, methods, voluntary nature of participation, right to withdraw consent, and confidentiality protections. Paper consent forms and questionnaires were stored in a locked cabinet at Company B. Electronic data were password-protected and managed by the researcher on a USB drive kept in a locked desk at the Kanazawa University.

## Results

Participant characteristics

Overall, 613 male workers were included in the final analysis. The mean participant age was 39.9 ± 12.6 years. By job type, 415 (67.7%) and 198 (32.3%) participants were blue- and white-collar workers, respectively. The proportions classified as meeting the criteria for metabolic and borderline metabolic syndromes were 78 (12.7%) and 90 (14.7%), respectively, totaling 168 (27.4%). The mean serum uric acid level was 5.9 ± 1.2 mg/dL, and 126 participants (20.6%) were classified into the high uric acid group (serum uric acid level: ≥7.0 mg/dL; Table 1).

**Table 1:** Characteristics of all participants (n=613) BMI: body mass index; eGFR: estimated glomerular filtration rate; LDL: low-density lipoprotein; HDL: high-density lipoprotein; AST: aspartate aminotransferase; ALT: alanine aminotransferase; γ-GTP: gamma-glutamyl transferase; HbA1c: glycated hemoglobin.

Variable		n (%)	Mean ± SD
Sex	Male	613 (100)	
Age (years)			39.9 ± 12.6
Household composition	Living alone	108 (17.6)	
	Living with others	490 (79.9)	
	Other	15 (2.4)	
Job type	White-collar (sales, clerical, and design)	198 (32.3)	
	Blue-collar (factory workers)	415 (67.7)	
Antihypertensive medication use	Yes	59 (9.6)	
	No	554 (90.4)	
Hypoglycemic medication or insulin use	Yes	16 (2.6)	
	No	597 (97.4)	
Lipid-lowering medication use	Yes	33 (5.4)	
	No	580 (94.6)	
Metabolic syndrome classification	Met criteria	78 (12.7)	
	Borderline	90 (14.7)	
	Not met	445 (72.6)	
Serum uric acid (mg/dL)			5.9 ± 1.2
Height (cm)			171.0 ± 5.7
Body weight (kg)			68.7 ± 11.5
BMI (kg/m²)			23.4 ± 3.6
Waist circumference (cm)			82.3 ± 10.2
Systolic blood pressure (mmHg)			122.8 ± 16.3
Diastolic blood pressure (mmHg)			75.2 ± 12.3
Creatinine (mg/dL)			0.9 ± 0.1
eGFR (mL/min/1.73m²)			83.5 ± 14.9
Total cholesterol (mg/dL)			191.8 ± 35.0
LDL cholesterol (mg/dL)			111.5 ± 30.6
HDL cholesterol (mg/dL)			59.5 ± 15.2
AST (U/L)			22.5 ± 10.0
ALT (U/L)			25.5 ± 18.5
γ-GTP (U/L)			31.5 ± 26.5
HbA1c (%)			5.6 ± 0.4

Comparison of baseline characteristics between the groups

The normal and high uric acid groups comprised 487 (79.4%) and 126 (20.6%) participants, respectively. The corresponding mean participant ages were 40.2 ± 12.7 and 38.5 ± 12.2 years. The high uric acid group tended to be younger; however, the between-group difference in age was not statistically significant. No significant between-group differences were found in household composition or medication use (antihypertensive, hypoglycemic, or lipid-lowering agents; Table 2).

**Table 2:** Comparison of baseline characteristics between the normal and high uric acid groups ^a^: Pearson's chi-squared test; ^b^: Student's t-test; —: statistical test not performed due to a cell count of zero. BMI: body mass index; eGFR: estimated glomerular filtration rate; LDL: low-density lipoprotein; HDL: high-density lipoprotein; AST: aspartate aminotransferase; ALT: alanine aminotransferase; γ-GTP: gamma-glutamyl transferase; HbA1c: glycated hemoglobin.

Variable		Normal group (n=487)		High uric acid group (n=126)		P value	
		n (%)	Mean ± SD	n (%)	Mean ± SD		
Sex	Male	487 (100)		126 (100)			
Age (years)			40.2 ± 12.7		38.5 ± 12.2	0.172	^b^
Household composition	Living alone	85 (17.5)		23 (18.3)		0.401	^a^
	Living with others	388 (79.5)		103 (80.9)			
	Other	14 (2.9)		1 (0.8)			
Antihypertensive medication use	Yes	47 (9.7)		12 (9.5)		0.966	^a^
	No	440 (90.3)		114 (90.5)			
Hypoglycemic medication or insulin use	Yes	16 (3.3)		0 (0)		-	
	No	471 (96.7)		126 (100)			
Lipid-lowering medication use	Yes	29 (6.0)		4 (3.2)		0.218	^a^
	No	458 (94.0)		122 (96.8)			
Metabolic syndrome classification	Met criteria	55 (11.3)		23 (18.3)		0.002	^a^
	Borderline	63 (12.9)		27 (21.4)			
	Not met	369 (75.8)		76 (60.3)			
Height (cm)			171.1 ± 5.6		170.4 ± 5.8	0.172	^b^
Body weight (kg)			67.8 ± 11.3		72.5 ± 11.6	<0.001	^b^
BMI (kg/m²)			23.1 ± 3.4		25.0 ± 3.8	<0.001	^b^
Waist circumference (cm)			81.3 ± 10.0		86.2 ± 10.4	<0.001	^b^
Systolic blood pressure (mmHg)			121.8 ± 16.2		126.8 ± 16.2	0.002	^b^
Diastolic blood pressure (mmHg)			74.7 ± 12.3		77.5 ± 12.0	0.023	^b^
Creatinine (mg/dL)			0.8 ± 0.1		0.9 ± 0.1	0.001	^b^
eGFR (mL/min/1.73m²)			84.1 ± 14.9		81.3 ± 14.5	0.067	^b^
Total cholesterol (mg/dL)			189.8 ± 34.8		199.9 ± 34.5	0.004	^b^
LDL cholesterol (mg/dL)			109.8 ± 30.2		118.2 ± 31.1	0.006	^b^
HDL cholesterol (mg/dL)			60.1 ± 14.7		57.3 ± 16.9	0.098	^b^
AST (U/L)			21.4 ± 9.3		25.7 ± 11.6	<0.001	^b^
ALT (U/L)			22.8 ± 12.3		36.2 ± 30.7	<0.001	^b^
γ-GTP (U/L)			29.4 ± 25.3		39.7 ± 29.6	<0.001	^b^
HbA1c (%)			5.6 ± 0.5		5.6 ± 0.3	0.603	^b^

All anthropometric measurement values were significantly higher in the high uric acid group: body weight, 67.8 ± 11.3 kg vs. 72.5 ± 11.6 kg; BMI, 23.1 ± 3.4 kg/m² vs. 25.0 ± 3.8 kg/m²; and waist circumference, 81.3 ± 10.0 cm vs. 86.2 ± 10.4 cm (p<0.001 for all). Systolic blood pressure (121.8 ± 16.2 mmHg vs. 126.8 ± 16.2 mmHg, p=0.002) and diastolic blood pressure (74.7 ± 12.3 mmHg vs. 77.5 ± 12.0 mmHg, p=0.023) were also significantly higher in the high uric acid group, as was the distribution of metabolic syndrome classification (Met criteria: n=55 (11.3%) vs. n=23 (18.3%); Borderline: n=63 (12.9%) vs. n=27 (21.4%); Not met: n=369 (75.8%) vs. n=76 (60.3%); p=0.002).

Regarding clinical laboratory values, total cholesterol (189.8 ± 34.8 mg/dL vs. 199.9 ± 34.5 mg/dL, p=0.004) and LDL cholesterol (109.8 ± 30.2 mg/dL vs. 118.2 ± 31.1 mg/dL, p=0.006) were significantly higher in the high uric acid group, whereas HDL cholesterol did not differ significantly between the groups. Furthermore, the levels of all liver function markers were significantly higher in the high uric acid group, with particularly large between-group differences observed for ALT and γ-GTP: AST, 21.4 ± 9.3 U/L vs. 25.7 ± 11.6 U/L (p<0.001); ALT, 22.8 ± 12.3 U/L vs. 36.2 ± 30.7 U/L (p<0.001); and γ-GTP, 29.4 ± 25.3 U/L vs. 39.7 ± 29.6 U/L (p<0.001). Creatinine was significantly higher in the high uric acid group (0.8 ± 0.1 mg/dL vs. 0.9 ± 0.1 mg/dL, p=0.001), whereas eGFR tended to be lower in this group without reaching statistical significance. HbA1c did not differ significantly between the groups.

Comparison of workplace environment and lifestyle factors between the groups

No significant between-group differences were found in job type, years of service, or daily working hours. Regarding lunch type, the proportion of participants who used the company cafeteria was significantly higher in the normal group (n=139; 28.4%) than in the high uric acid group (n=20; 15.9%; p=0.004). The proportion of participants who most frequently consumed tea during work hours tended to be higher in the high uric acid group (n=66; 52.4%) than in the normal group (n=209; 42.9%), although this difference did not reach statistical significance (p=0.057; Table 3).

**Table 3:** Comparison of workplace environmental factors between the normal and high uric acid groups ^a^: Pearson's chi-squared test; ^b^: Student's t-test.

Variable		Normal group (n=487)		High uric acid group (n=126)		P value	
		n (%)	Mean ± SD	n (%)	Mean ± SD		
Job type	White-collar (sales, clerical, and design)	159 (32.6)		39 (31.0)		0.717	^a^
	Blue-collar (factory workers)	328 (67.4)		87 (69.0)			
Years of service (years)			16.2 ± 12.1		14.8 ± 11.3	0.247	^b^
Daily working hours (hours/day)			9.2 ± 1.2		9.2 ± 1.2	0.817	^b^
Lunch type (multiple responses)	Company cafeteria	139 (28.5)		20 (15.9)		0.004	^a^
	Supermarket	35 (7.2)		5 (4.0)		0.192	^a^
	Convenience store	55 (11.3)		15 (11.9)		0.848	^a^
	Restaurant	5 (1.0)		2 (1.6)		0.598	^a^
	Self-prepared bento	60 (12.3)		22 (17.5)		0.131	^a^
	Family-prepared bento	179 (36.8)		55 (43.7)		0.156	^a^
	Skipping lunch	17 (3.5)		7 (5.6)		0.287	^a^
Cafeteria use frequency (times/week)			4.5 ±1.1		4.1 ±1.5	0.168	^b^
Type of beverage consumed during work (multiple responses)	Water	118 (24.2)		26 (20.6)		0.396	^a^
	Tea	209 (42.9)		66 (52.4)		0.057	^a^
	Sports drink	19 (3.9)		3 (2.4)		0.413	^a^
	Juice	24 (4.9)		6 (4.8)		0.939	^a^
	Coffee	130 (26.7)		27 (21.4)		0.227	^a^

Regarding lifestyle factors, a significant between-group difference was observed in smoking status (p=0.048), with a higher proportion of former smokers in the high uric acid group (n=34; 27%) than in the normal group (n=87; 17.9%). Weight gain of ≥10 kg since the age of 20 years was significantly more prevalent in the high uric acid group (n=55; 43.7%) than in the normal group (n=160; 32.9%; p=0.024). The proportion with regular exercise habits was significantly lower in the high uric acid group (n=23; 18.3%) than in the normal group (n=137; 28.1%; p=0.024). The proportion skipping breakfast three or more times per week was significantly higher in the high uric acid group (n=45; 35.7%) than in the normal group (n=118; 24.2%; p=0.009). No significant between-group differences were found in alcohol consumption frequency, eating dinner within two hours of bedtime, or sleep adequacy (Table 4).

**Table 4:** Comparison of lifestyle factors between the normal and high uric acid groups Pearson's chi-squared test.

Variable		Normal group (n=487)		High uric acid group (n=126)		P value
		n	%	n	%	
Smoking status	Current smoker	200	41.1	51	40.5	0.048
	Former smoker (quit within past month)	87	17.9	34	27.0	
	Never smoker	200	41.1	41	32.5	
Weight gain ≥10 kg since age 20	Yes	160	32.9	55	43.7	0.024
	No	327	67.1	71	56.3	
Regular exercise habits (≥30 min, ≥2 times/week, ≥1 year)	Yes	137	28.1	23	18.3	0.024
	No	350	71.9	103	81.7	
Daily walking ≥1 hour	Yes	248	50.9	58	46.0	0.328
	No	239	49.1	68	54.0	
Eating dinner within 2 hours of bedtime ≥3 times/week	Yes	231	47.4	48	38.1	0.061
	No	256	52.6	78	61.9	
Snacking or consuming sweet beverages	Daily	98	20.1	24	19.0	0.927
	Sometimes	310	63.7	80	63.5	
	Rarely	79	16.2	22	17.5	
Skipping breakfast ≥3 times/week	Yes	118	24.2	45	35.7	0.009
	No	369	75.8	81	64.3	
Alcohol consumption frequency	Daily	105	21.6	33	26.2	0.394
	Sometimes	204	41.9	54	42.9	
	Never	178	36.6	39	31.0	
Adequate sleep	Yes	252	51.7	63	50.0	0.727
	No	235	48.3	63	50.0	

Factors independently associated with hyperuricemia risk

The results of multivariable logistic regression analysis, in which variables with a p-value of <0.1 in the univariable analyses were entered as independent variables, are shown in Table 5.

**Table 5:** Factors independently associated with elevated serum uric acid (multivariable logistic regression, n=613) OR: odds ratio; CI: confidence interval; eGFR: estimated glomerular filtration rate; ALT: alanine aminotransferase. Multivariable logistic regression with stepwise variable elimination. For continuous variables, OR represents the odds ratio per 1-unit increase.

Variable		OR	95% CI		P value
			Lower	Upper	
Company cafeteria use	Yes	0.528	0.304	0.919	0.024
	No (reference)	1.000			
Tea as primary beverage during work	Yes	1.507	0.977	2.324	0.064
	No (reference)	1.000			
Smoking (current or former)	Yes	1.861	1.124	3.08	0.016
	No (reference)	1.000			
Regular exercise habits	Yes	0.501	0.288	0.871	0.014
	No (reference)	1.000		—	
Eating dinner within 2 hours of bedtime ≥3 times/week	Yes	0.647	0.412	1.017	0.059
	No (reference)	1.000			
Skipping breakfast ≥3 times/week	Yes	1.684	1.036	2.737	0.035
	No (reference)	1.000			
Age (years)	Continuous	0.952	0.929	0.976	<0.001
Systolic blood pressure (mmHg)	Continuous	1.014	1.000	1.028	0.052
eGFR (mL/min/1.73m²)	Continuous	0.969	0.950	0.988	0.001
ALT (U/L)	Continuous	1.035	1.022	1.048	<0.001

Factors significantly associated with increased odds of being in the high uric acid group were smoking (odds ratio or OR: 1.861, 95% confidence interval or CI: 1.124-3.080), skipping breakfast three or more times per week (OR: 1.684, 95% CI: 1.036-2.737), and ALT (OR: 1.035, 95% CI: 1.022-1.048). The factors significantly associated with reduced odds were company cafeteria use (OR: 0.528, 95% CI: 0.304-0.919), regular exercise habits (OR: 0.501, 95% CI: 0.288-0.871), age (OR: 0.952, 95% CI: 0.929-0.976), and eGFR (OR: 0.969, 95% CI: 0.950-0.988). Tea consumption during work hours, eating dinner within two hours of bedtime, and systolic blood pressure remained in the final model but did not reach statistical significance.

## Discussion

In this study, 126 (20.6%) of the participants were classified into the high uric acid group. This proportion was slightly lower than the prevalence of 26.8% reported in a large-scale Japanese study [22], likely because that study included individuals receiving pharmacological treatment for hyperuricemia, whereas the present study excluded treated individuals. The mean age of the high uric acid group (38.5 years) was slightly lower than that of the normal group (40.2 years). In Japan, the prevalence of hyperuricemia peaks among individuals in their 30s and 40s and appears to decline thereafter, partly because the number of individuals initiating urate-lowering therapy increases with age [22]. Given that early-onset hyperuricemia is a predictor of future cardiovascular disease, mortality, and chronic kidney disease [7,8], early intervention is of particular importance.

The high uric acid group showed significantly higher values than the normal group across all anthropometric measures (body weight, BMI, and waist circumference), blood pressure, lipid markers (total and LDL cholesterol), liver function markers (AST, ALT, and γ-GTP), and creatinine, as well as a significantly higher prevalence of metabolic syndrome. Multivariable analysis revealed ALT and eGFR to be independent correlates of uric acid, consistent with prior reports [9,10]. Although individual markers remained within the borderline or mildly elevated range, the high uric acid group showed significantly higher values across multiple metabolic indicators than those shown by the normal group.

Company cafeteria use was identified as a significant independent factor associated with reduced odds of being in the high uric acid group (OR: 0.528). The finding that workplace food environment is associated with serum uric acid levels represents a novel contribution, suggesting the potential effectiveness of environmental approaches to prevention that do not rely solely on individual behavioral change. At Company B, all cafeteria menu items displayed calorie information, and cafeteria users visited on an average of more than four times per week. Prior studies have reported that calorie labeling in workplace cafeterias significantly reduces calorie intake at lunch [23], and sustained use of the company cafeteria may have contributed to appropriate food selection, reduced obesity, and metabolic risk, thereby suppressing the increase in uric acid levels. The reasons for company cafeteria use were not directly assessed; however, factors such as affordability, convenience, and proximity to the workplace may have encouraged workers to use the cafeteria regularly.

Skipping breakfast three or more times per week was identified as a significant independent risk factor for hyperuricemia (OR: 1.684). This finding is consistent with that of a large-scale Korean study [24] and indicates that breakfast skipping is associated with uric acid levels among Japanese male workers. Breakfast skipping has been reported to increase insulin resistance [25], and the present finding that the high uric acid group had significantly higher BMI, waist circumference, and metabolic syndrome prevalence suggests that breakfast skipping may contribute to elevated uric acid levels through obesity and metabolic abnormalities. The reasons for breakfast skipping were not directly assessed in this study; however, a national survey on dietary education conducted by the Ministry of Agriculture, Forestry and Fisheries of Japan reported that individuals who do not eat breakfast daily identified appetite in the morning, time to prepare breakfast, ability to wake up early, and established breakfast habits as the primary factors necessary for eating breakfast [26]. These findings suggest that breakfast skipping among workers may be influenced by multiple factors, including appetite, time constraints, disrupted sleep-wake cycles, and lack of established dietary habits.

Regular exercise has been identified as a significant independent factor associated with reduced odds of hyperuricemia (OR: 0.501). Previous studies have shown that regular aerobic exercise lowers uric acid through improvements in body weight and insulin sensitivity [18], consistent with the present findings. Given that the high uric acid group had significantly higher BMI and waist circumference, the lack of regular exercise may have contributed to elevated uric acid levels through obesity-related mechanisms.

Smoking was identified as a significant independent risk factor for hyperuricemia (OR: 1.861). Although the association between smoking and uric acid has not been fully elucidated, the present finding is consistent with recent large-scale epidemiological evidence [21]. Notably, the proportion of former smokers was higher in the high uric acid group (n=34; 27.0%) than in the normal group (n=87; 17.9%). Smoking impairs renal function [27], and reduced renal uric acid excretion resulting from smoking-related renal dysfunction may have contributed to hyperuricemia. The identification of eGFR as an independent correlate in the present study further supports this pathway.

The findings of the present study suggest that promoting company cafeteria use through enhanced calorie labeling, supporting regular exercise habits, encouraging breakfast consumption, and providing smoking cessation support are specific priorities for reducing hyperuricemia risk among male manufacturing workers. The identification of company cafeteria use as an independent protective factor supports the effectiveness of environmental interventions. In companies with on-site cafeterias, creating an environment that facilitates healthy food choices, such as enhanced calorie labeling and health-promoting menu options, is likely to be effective [28].

The independent associations of breakfast skipping, exercise, and smoking with uric acid suggest that targeting a single lifestyle factor is insufficient. Comprehensive and sustained workplace interventions combining health education on breakfast consumption, promotion of regular exercise habits, and smoking cessation support are recommended. Furthermore, uric acid measurement is not included as a mandatory item in periodic health checkups, and no systematic health guidance program equivalent to the specific health guidance program for metabolic syndrome is available for workers under 40 years of age. Given that the high uric acid group in this study was relatively young, proactive uric acid screening within periodic health checkups and early intervention targeting younger workers are important priorities for occupational health practice.

This study has some limitations. First, the cross-sectional design precludes causal inferences regarding the observed associations; longitudinal studies are needed to verify these findings. Second, detailed dietary information, such as caloric intake, nutritional balance, and purine content, was not assessed, limiting direct examination of the mechanisms linking company cafeteria use and breakfast skipping with uric acid levels. Third, unmeasured confounders, including occupational stress, chemical exposure, and genetic factors, could not be accounted for. Fourth, lifestyle data were based on self-reported questionnaire responses, which may be subject to recall bias and social desirability bias, potentially affecting the accuracy of the findings. Fifth, participants were limited to male workers at a single manufacturing company without night shifts, and caution is warranted when generalizing these findings to other industries, work arrangements, or female workers.

## Conclusions

This study demonstrated that several male workers at a single manufacturing company in Japan met the criteria for hyperuricemia, and that this group showed significantly higher values across multiple indicators of obesity, hypertension, dyslipidemia, liver dysfunction, and impaired renal function compared with the normal group. Multivariable logistic regression analysis revealed that company cafeteria use as an independent workplace environmental factor, and exercise habits, breakfast skipping, and smoking as independent lifestyle factors, were each independently associated with hyperuricemia risk.

These findings suggest that promoting company cafeteria use through enhanced calorie labeling, supporting regular exercise habits, encouraging breakfast consumption, and providing smoking cessation support are specific priorities for reducing hyperuricemia risk among male manufacturing workers. Comprehensive and sustained workplace interventions combining health education on breakfast consumption, promotion of regular exercise habits, and smoking cessation support are recommended. Given that the high uric acid group was relatively young, proactive uric acid screening in periodic health checkups and early intervention for younger workers represent important priorities for future occupational health practice. Furthermore, longitudinal studies examining the long-term outcomes of hyperuricemia among workers, such as gout development and cardiovascular events, are warranted. Future interventional studies examining whether workplace interventions targeting cafeteria use, exercise habits, breakfast consumption, and smoking cessation effectively reduce uric acid levels among workers are also warranted.

## Appendices

Appendix A

Part 1: Medical Interview Items Administered at the Periodic Health Checkup of Company B

(1) Are you currently taking antihypertensive medication?※1 ・Yes ・No

(2) Are you currently using insulin injections or hypoglycemic medication?※1 ・Yes ・No

(3) Are you currently taking cholesterol※2- or triglyceride-lowering medication?※1 ・Yes ・No

(4) Has your body weight increased by 10 kg or more since the age of 20? ・Yes ・No

(5) Do you engage in light exercise (enough to break a sweat) for 30 minutes or more per session, at least twice a week, for at least 1 year? ・Yes ・No

(6) In your daily life, do you walk or engage in equivalent physical activity for at least 1 hour per day? ・Yes ・No

(7) Do you eat dinner within 2 hours of bedtime three or more times per week? ・Yes ・No

(8) Do you consume snacks or sweet beverages outside of your three main meals (breakfast, lunch, and dinner)? ・Every day ・Sometimes ・Rarely

(9) Do you skip breakfast three or more times per week? ・Yes ・No

(10) How often do you drink alcohol? ・Every day ・Sometimes ・Never (or unable to drink)

(11) Do you get adequate rest from sleep? ・Yes ・No

(12) Do you currently smoke habitually? (A habitual smoker is defined as someone who has smoked a total of 100 or more cigarettes or has smoked for 6 months or more, and has smoked within the past month.) ・Yes ・No → If "No": Have you quit smoking within the past month? ・Yes ・No

※1: Refers to individuals currently taking medication under the diagnosis and treatment of a physician. ※2: Triglycerides are treated in the same manner.

Appendix B

Part 2: Original Questionnaire on Workplace Environment and Lifestyle Habits

(1) What is your job type? ・White-collar (sales, clerical, or design) ・Blue-collar (primarily factory work)

(2) How many years have you worked at this company? ___ years

(3) How many hours per day do you work on average? (Please answer based on the past month.) ___ hours ___ minutes per day

(4) How do you usually have lunch at work? (Please select the most common option.) ・Company cafeteria ・Food purchased at a supermarket ・Food purchased at a convenience store ・Eating out ・Bento prepared by myself ・Bento prepared by family or others

(5) For those who use the company cafeteria: How often do you use the company cafeteria? ___ times per week

(6) What type(s) of beverage do you consume during work? ・Water ・Tea ・Sports drink ・Juice ・Coffee

(7) Sex ・Male ・Female

(8) Household composition (Please select one.) ・Living alone ・Living with spouse ・Living with spouse and children ・Living with children only ・Living with parent(s) ・Living with parent(s) and spouse ・Living with parent(s), spouse, and children ・Living with parent(s) and children ・Other
